# Assessment of Parents’ Perceptions of Childhood Immunization: A Cross-Sectional Study from Pakistan

**DOI:** 10.3390/children8111007

**Published:** 2021-11-04

**Authors:** Azhar Hussain, Anam Zahid, Madeeha Malik, Mukhtar Ansari, Mojtaba Vaismoradi, Adeel Aslam, Khezar Hayat, Márió Gajdács, Shazia Jamshed

**Affiliations:** 1 Department of Pharmacy Practice, Hamdard Institute of Pharmaceutical Sciences, Hamdard University, Islamabad 45550, Pakistan; azharhussain26@hotmail.com (A.H.); anamzahid4@gmail.com (A.Z.); ceo@cyntaxhealthprojects.com (M.M.); 2 Department of Clinical Pharmacy, College of Pharmacy, University of Hail, Hail 81451, Saudi Arabia; mukhtaransari@hotmail.com; 3 Faculty of Nursing and Health Sciences, Nord University, 8049 Bodø, Norway; mojtaba.vaismoradi@nord.no; 4 Department of Pharmacy Practice, Kulliyyah of Pharmacy, International Islamic University Malaysia, Kuantan 25200, Pahang, Malaysia; adeelaslam224@gmail.com; 5 Institute of Pharmaceutical Sciences, University of Veterinary and Animal Sciences, Lahore 54000, Pakistan; Khezar.hayat@uvas.edu.pk; 6 Department of Oral Biology and Experimental Dental Research, Faculty of Dentistry, University of Szeged, Tisza Lajos körút 63, 6720 Szeged, Hungary; 7 Institute of Medical Microbiology, Faculty of Medicine, Semmelweis University, Nagyvárad tér 4, 1089 Budapest, Hungary; 8 Department of Clinical Pharmacy and Practice, Faculty of Pharmacy, Universiti Sultan Zainal Abidin, Kuala Terengganu 21300, Malaysia; pharmacist1992@live.com; 9 Qualitative Research-Methodological Application in Health Sciences Research Group, Kulliyyah of Pharmacy, International Islamic University Malaysia, Kuantan 25200, Pahang, Malaysia

**Keywords:** children, immunization, knowledge, attitudes, perceptions, assessment, Pakistan

## Abstract

Immunization is one of the most cost-effective public health interventions, with considerable impacts on people’s health. Parents’ perception of their knowledge, attitude, and satisfaction is an important factor, as they may be targeted by interventions for better immunization coverage. Therefore, this study aimed to assess parents’ perceptions in terms of their knowledge, attitude, and satisfaction of the immunization of their children aged less than two years of age, in two cities of Pakistan. A descriptive cross-sectional study was conducted in the vicinity of Rawalpindi and Islamabad from March to August 2019. A semi-structured questionnaire was used for the data collection on a convenient sample of parents. The questionnaire was hand-delivered to the parents by data collectors. Descriptive and inferential statistics were used for data analysis via SPSS version 22. A total of *n* = 382 respondents were included in the data analysis. Statistically significant differences were found between the parents’ knowledge scores and their education levels and monthly incomes (*p* < 0.05). Parents with master’s education degrees and low monthly incomes had significantly better knowledge (*p* < 0.05). Additionally, 96.85% of the respondents believed that child immunization was important. In addition, more than half of the respondents (57.58%) thought that the affordability of vaccines was a principal factor for delays in immunization. Although the parents’ knowledge regarding the immunization of their children was not adequate, they had positive perceptions toward it.

## 1. Introduction

Immunization is one of the most cost-effective public health interventions, with considerable impacts on people’s health [1]. It is quite fortunate that each year, the lives of more than a million people (especially very young children) around the globe are saved with the help of immunization. However, at the same time, every fifth child born on the planet misses the chance of being immunized and thus reducing their chance to live past 5 years of age [2]. The achievement in the high rates of immunization coverage in many countries has been one of the greatest efforts of public health; improving the coverage of childhood vaccination is a key health policy objective in many developing countries. Parents’ knowledge and attitudes have a significant influence on the immunization rates of their children. Increasing the immunization coverage to above 80% should cut the chain of disease transmission and provide herd immunity in a specific area. To achieve this goal, a full course of appropriate vaccines should be given at the right time [3]. However, there is a great difference between immunization rates in developing/transitional versus developed countries [4,5,6]. According to global health data, immunization saves the life of nearly three million people each year [7]. About 30 million children in Latin America, sub-Saharan Africa, and Asia still do not have access to basic immunization services, due to which a child dies from a vaccine-preventable disease every ten seconds [8]. Therefore, immunization is a method to prevent children from dying of infectious childhood diseases.

A look at the infant mortality rate (IMR) of developed as well as developing/transitional countries (including Pakistan), shows an increased incidence of mortality and morbidity among children globally. Therefore, increasing the general population’s access to immunization services may reduce IMR at large [9]. However, to meet the goal of increased immunization coverage, barriers to immunization should be identified and addressed [7]. Various studies performed in different countries indicate that variations in the barriers to vaccination, and thus under-immunization, may occur due to multiple mechanisms. A study in the USA showed that socioeconomic factors, traditional factors (i.e., consulting shamans and herbalists) and the nativity of the population were major barriers to immunization [10]. A study performed in England reported parents’ refusal to get their children vaccinated as they feared vaccines’ safety levels [11]. In some African countries, including Nigeria, lack of parental knowledge and negative attitudes regarding immunization in children are the most important barriers. Mothers may also be fearful of vaccine-related complications and adverse events [12]. These barriers may be addressed by increasing access to immunization services and by providing evidence-based and authentic medical information for the public. Up-to-date immunization coverage is essential for protecting every age group from debilitating and potentially life-threatening consequences of severe infectious diseases. Our understanding of the current statistics of immunized children, and the quality of immunization services in Pakistan is limited. Therefore, this study aimed to assess parents’ perceptions in terms of their knowledge, attitude, and satisfaction of the immunization of their children aged less than two years of age in two cities of Pakistan.

## 2. Materials and Methods

### 2.1. Study Site, Setting

A descriptive cross-sectional study was conducted in the immunization centers of tertiary care hospitals (*n* = 15), secondary care hospitals (*n* = 29), basic health units, and dispensaries having immunization units for child vaccination (*n* = 98), situated in the vicinity of Islamabad and Rawalpindi [13,14]. Respondents included in the current study were parents of the Muslim faith, having children aged less than two years of age, and residing in the vicinity of Rawalpindi and Islamabad (capital city) in Pakistan.

### 2.2. Sample Size and Sampling Procedure

The Raosoft^®^ Sample Size Calculator was used to determine the required sample size needed for this study [15]. The calculated sample size was *n* = 382 people, given a 95% confidence level and 5% margin of error. A convenience sampling technique was used to select the respondents from the study sites.

### 2.3. Data Collection Tool and Its Development

The first draft of the semi-structured questionnaire was developed through an extensive review of the literature and by using the Pakistan Expanded Programme on Immunization (EPI) childhood immunization guidelines. Then, the second draft of the questionnaire was developed through focus group discussions. Two focus group discussions were held at different time intervals with four different groups of experts, including clinicians, specialists, physicians, and doctors from academia. The questionnaire was comprised of four sections as follows: (i) respondent demographic characteristics such as gender, age, occupation, qualification, and the total number of children; (ii) parents’ perceptions regarding the significance of immunization, religious beliefs, timing of vaccinations, factors affecting immunization practices, an awareness campaign about immunization, and activeness of the awareness campaign in Pakistan. A set of statements was also included in the questionnaire in which respondents were asked to indicate their level of agreement using a 5-point Likert scale; (iii) parents’ knowledge regarding the Pakistan EPI childhood immunization schedule for children under two years of age. The composite score range was 14–28, and a lower score indicated better knowledge. Each correct response for question marked one and incorrect response marked two and then total scores were calculated based on these responses; (iv) the satisfaction of parents regarding immunization services in Pakistan.

Data were collected by previously trained data collectors who hand-delivered the questionnaire to the parents. Respondents were assured about the confidentiality of information verbally and were shown an undertaking signed by the principal investigator. The questionnaire was self-completed by the respondents and was collected from them on the same day.

### 2.4. Reliability and Validity of the Tool

A panel of experts (*n* = 8) consisting of clinicians, specialists, physicians, and doctors from academia discussed and appraised both the content and face validity of the final version of the questionnaire. Pilot testing was carried out on 38 respondents (10%) of the total sample size before the execution of the final study. A Cronbach alpha value of 0.76 confirmed the reliability and internal consistency of the questionnaire. The study was approved by the Ethical Committee of Hamdard University (Ref# 638).

### 2.5. Data Analysis

Collected data were coded and entered in the SPSS statistics software, version 22. To check the normal distribution of data, skewness and kurtosis tests were performed. Descriptive statistics of frequency and percentage were calculated. The Mann‐Whitney test (*p* ≤ 0.05) was performed to identify differences among variables.

## 3. Results

### 3.1. Socio-Demographic Characteristics of the Respondents

Out of *n* = 382 respondents, *n* = 213 (55.76%) were male. Nearly two percent (*n* = 1.92%) of them had no formal education; 3.60% had education up to the primary level, 15.44% had education up to the secondary, and 5.23% had completed their higher secondary education. About one-third of the respondents (37.95%) were graduates, and 35.86% of them had education up to the master (MSc/MA) level in different fields.

When it comes to employment, 11.25% of the respondents were working in government organizations, 7.85% in the healthcare sector, 19.37% in the private sector, 11.78% were entrepreneurs/running their own business, 17.54% in the education sector, 3.14% were blue-collar workers, 2.36% were bankers, lawyers, and religious scholars, and 22.51% did not mention their workplace/affiliation. Regarding the number of children, 27.48% had a single child, 33.25% had two children, and 21.46% had three children (Table 1).

### 3.2. Parents’ Perceptions about Immunization of Their Children under Two Years of Age

The majority of the respondents (*n* = 370, 96.85%) believed that child immunization was important, while *n* = 348 (91.1%) agreed that immunization was safe for children. Considering the religious point of view, *n* = 98 (25.65%) of the respondents thought that immunization was prohibited by their religion (Table 2).

### 3.3. Parents’ Perceptions Regarding Immunization Services and Factors Responsible for Delay in the Immunization of Their Children

Around two-thirds of respondents (*n* = 255, 66.74%) were satisfied with the immunization services offered by the government. Furthermore, *n* = 335 (87.69%) of them agreed that an awareness campaign about childhood immunization was effective and reached the community. In addition, *n* = 279 (73.00%) agreed that an awareness campaign about childhood immunization was active in Pakistan (Table 3). Affordability (57.5%), costly vaccines or their unavailability at the hospitals on visits (53.0%), non-cooperative staff (37.4%), long waiting time for the vaccination of children (42.0%), lack of awareness regarding immunization timing (78.0%), and lack of access to immunization centers (58.6%) were the principal factors contributing to delay in the immunization of children, based on our surveyed population (Table 4).

### 3.4. Knowledge of Parents Regarding Immunization and Its Schedule for Their Children under Two Years of Age

The respondents had a different level of knowledge regarding children’s immunization schedules. Accordingly, 10.98% of them know that vaccines should be given to children at birth. While 10.46% knew that vaccines should be given to children during twelve to twenty-three months of age (13.87%), followed by vaccines given at nine months of age (12%), vaccines given at the age of the tenth week (8.37%), and vaccines given at fourteen weeks of age (8.37%). The results also highlighted that 44.23% of them were aware of the immunization day. They (37%) agreed that their children experienced adverse events due to immunization. They mostly (69.37%) were aware of the mobile immunization team. A detailed description of parents’ knowledge regarding immunization schedules for their children under two years of age is illustrated in Table 5.

### 3.5. Parents’ Satisfaction with Immunization Services Offered in Pakistan

A total of 56% of the respondents were satisfied with the services provided by the mobile immunization team, and 44% were dissatisfied. Furthermore, 73.56% were satisfied with the immunization provided at hospital centers. A detailed description of parents’ satisfaction with immunization services offered in Pakistan has been illustrated in Table 6.

### 3.6. Measures to Be Taken for the Improvement of Immunization Services in Pakistan

Regarding measures for improving immunization services in Pakistan, 26.17% of the respondents suggested that promoting awareness and training of parents can improve immunization services in Pakistan. While 5.75% of respondents said easy availability of vaccines followed by accessibility to immunization centers, proper vaccine management system/health system (8.90%), improvement of services of mobile immunization team, availability of mobile immunization team (6.54%), and improvement of vaccination services (3.92%) were important methods to improve immunization services in Pakistan (Table 7).

### 3.7. Comparison of Parents’ Knowledge Scores about the Immunization of Their Children under Two Years of Age by Demographic Characteristics

The composite scores (mean ranks) for knowledge were taken into account when assessing the knowledge of parents by using the Mann–Whitney test. Significant differences (*p* ≤ 0.05) were found among the knowledge scores of parents with different qualifications and monthly incomes. Parents with a master’s degree and having monthly incomes between Rs 20,000 and 30,000 had significantly better knowledge than others. While no significant differences (*p* ≤ 0.05) were found between the genders, age, occupation, city, and the number of children (Table 8).

## 4. Discussion

Parents play a key role in the immunization of their children, their knowledge and attitude towards vaccination, and their satisfaction with national immunization services have a significant influence on immunization coverage. In the current study, the parents did not have sufficient knowledge regarding immunization schedules, although they received immunization cards from the hospitals or immunization centers. It is believed that parental knowledge plays an important role in the success of immunization programs. The results of the present study showed that the respondents were mostly aware of the importance of immunization. Similar findings were reported from the studies of other authors conducted in Pakistan and the UK [16,17]. Mostly Muslim parents stated that religion had a prohibitory role in the participation of immunization, and so they were among the highest non-vaccinated families, and their household also had lesser complete coverage of vaccination than the other religious communities [18].

Timing and consequences of immunization influence people’s attitudes toward immunization. Nearly half of the participants agreed that their children experienced short-term side effects after taking vaccines. The respondents also believed that vaccination timing matters and they are likely to follow the immunization schedule. The findings are in line with another study carried out in Pakistan indicating the occurrence of side effects such as rashes, pain, fever, and swelling after immunization. Additionally, some people practiced the reversion of communicable diseases even though the vaccination course was complete [17].

The result of the present study highlighted that affordability, unavailability of vaccines in immunization centers at the time of vaccination, access to the immunization centers, long waiting times, the non-cooperative attitude of the medical staff, and lack of awareness regarding immunization timings as the main contributing factors to delays in immunization. Similarly, logistical problems, the attitude of the healthcare staff during the campaign of immunization, and long waiting times have been reported as barriers in Colombia and the USA [19,20,21]. The present study showed the inadequate knowledge of parents regarding the vaccination schedule of Bacille Calmette‐Guerin (BCG), polio, measles, diphtheria, tetanus toxoids, and pertussis (DPT) vaccine. They did not consider it important for child safety. They failed to realize the benefits associated with immunization services. Published studies from India and Pakistan also reported a similar inadequate level of knowledge among parents regarding immunization [22,23].

More than half of the respondents reported satisfaction with services delivered by the mobile immunization team. Another study in Pakistan reported that mobile immunization team services enhanced the immunization coverage and also increased the awareness in people about diseases which could be prevented through vaccination [24]. In our study, the majority of the parents were not aware of the proper immunization schedule, but they were satisfied with the immunization services offered by the government of Pakistan. This might be due to a lack of awareness of appropriate immunization practices among parents. The results were contradictory to those of the study conducted in the UK, in which the majority of parents had serious concerns about practice offered by the government [25]. If all vaccines provided in the hospital would be free of cost in Pakistan, it would help increase immunization coverage because family income plays an important role in availing free healthcare services like immunization [26].

The results of the present study showed that more than half of the respondents agreed that immunization services provided to children less than two years of age were free of charge in Pakistan. Furthermore, immunization activities are limited to urban areas only, whereas little attention is paid to rural areas. Similarly, a significant difference was reported between urban and rural mothers regarding the importance of vaccination and the age of initiation and completion of vaccination schedules in India [27].

Results from the current study showed that long waiting times and non-cooperative staff caused a delay in childhood immunization. Two other studies also showed that supportive staff, convenient office times, and limited waiting time for immunizations contributed to fully immunized children [28,29]. Another factor that was significantly associated with delay in childhood immunization was a lack of awareness regarding immunization (78%). Studies performed in Sudan, Kenya, and sub-Saharan Africa also showed that lack of awareness and information about childhood immunization were important contributors to the high burden of unimmunized children [30,31,32].

This study was carried out only in two cities of Pakistan on a convenient sample of parents. Given the limited resources and a limited time frame, we could not collect data on the general behaviors of Pakistani parents regarding immunization. While the results may not be generalized to other cities in Pakistan, they may reflect the general perception of people regarding immunization. Additionally, all aspects of EPI immunization guidelines were not assessed by the parents due to their busy routine and because females mostly were less educated, resulting in difficulties in understanding and following guidelines available to them in the form of immunization cards. Lastly, no test–retest was conducted, because in Pakistan mostly females come for vaccination and they do not have mobile phones and it was not possible to request them to give their home address for follow-up, because it is considered very bad in Pakistani culture.

## 5. Conclusions

The knowledge of parents regarding the immunization of children under two years of age was insufficient, but they had positive perceptions toward the immunization of children. Despite the availability of EPI immunization guidelines for parents in the form of immunization cards, they missed immunization schedules as they were unaware of the schedule and immunization timing of children. Immunizing their children just to follow others and without understanding the importance of immunization was common.

There is a dire need to educate people about the importance of immunization and enhance their knowledge of diseases against which they must immunize their children. To increase awareness and knowledge about vaccines and vaccine-preventable diseases, a collaboration of the government, as well as healthcare professionals, is deemed of importance. Awareness improving programs should be launched, focusing on people with less or no formal education. Strengthening the service of the mobile immunization team through providing better incentives to them as well as their appropriate training should be conducted. Training sessions for parents regarding immunization should also be arranged.

## Figures and Tables

**Table 1:** Demographic characteristics of the respondents.

Characteristics		Rawalpindi *n* (%)	Islamabad *n* (%)	Composite *n* (%)
Gender	Male	71 (18.58)	142 (37.17)	213 (55.76)
	Female	76 (19.89)	93 (24.34)	169 (44.24)
Age, yrs	Less than 20	2 (0.5)	2 (0.5)	4 (1.05)
	21–30	56 (14.65)	110 (18.79)	166 (43.45)
	31–40	73 (19.10)	88 (23.03)	161 (42.14)
	More than 40	13 (3.4)	38 (9.94)	51 (13.35)
Education	No formal education	2 (0.5)	5 (1.3)	7 (1.83)
	Primary education	8 (2.09)	6 (1.57)	14 (3.66)
	Secondary education	28 (7.32)	31 (8.11)	59 (15.44)
	Higher secondary education	4 (1.05)	16 (4.18)	20 (5.23)
	Graduate	51 (13.35)	94 (24.60)	145 (37.95)
	Master’s degree	51 (13.35)	86 (22.51)	137 (35.86)
Monthly income, Rs	Less than 10,000	5 (1.30)	8 (2.09)	13 (3.40)
	10,000–20,000	24 (6.28)	36 (9.42)	60 (15.70)
	20,000–30,000	9 (2.35)	27 (7.06)	36 (9.42)
	30,000–40,000	17 (4.45)	36 (9.42)	53 (13.87)
	40,000–50,000	14 (3.66)	30 (7.85)	44 (11.51)
	More than 50,000	27 (7.06)	63 (16.49)	90 (13.56)
	No income source	48 (12.56)	38 (9.94)	86 (22.51)
Occupation	Governmental	11 (2.87)	32 (8.37)	43 (11.25)
	Healthcare professional	10 (2.6)	20 (5.23)	30 (7.85)
	Private employee	24 (6.28)	50 (13.08)	74 (19.37)
	Business	11 (2.8)	34 (8.9)	45 (11.78)
	Engineers	8 (2.09)	7 (1.83)	15 (3.93)
	Academia	20 (5.23)	47 (12.30)	67 (17.54)
	Blue collar	6 (1.57)	6 (1.57)	12 (3.14)
	Homemaker	48 (12.56)	38 (9.94)	86 (22.51)
	Banker/other professional	4 (1.04)	4 (1.04)	8 (2.10)
Number of children	One	40 (10.47)	65 (17.01)	105 (27.48)
	Two	46 (12.04)	81 (21.20)	127 (33.25)
	Three	29 (7.59)	53 (13.87)	82 (21.46)
	More than three	29 (7.59)	39 (10.20)	68 (17.80)

**Table 2:** Parents’ perceptions about the immunization of their children under two years of age.

Questions	Islamabad			Rawalpindi			Composite		
	Strongly Agree/ Agree *n* (%)	Neutral *n* (%)	Strongly Disagree/ Disagree *n* (%)	Strongly Agree/ Agree *n* (%)	Neutral *n* (%)	Strongly Disagree/ Disagree *n* (%)	Strongly Agree/ Agree *n* (%)	Neutral *n* (%)	Strongly Disagree/ Disagree *n* (%)
Do you think childhood immunization is important?	228 (59.68)	10 (2.62)	Nil	142 (37.17)	2 (0.52)	Nil	370 (96.85)	12 (3.14)	Nil
Do you think childhood immunization is beneficial?	219 (57.33)	14 (3.66)	5 (1.30)	129 (33.77)	13 (3.40)	2 (0.52)	366 (95.81)	27 (7.06)	7 (1.83)
Do you think immunization is safe for children?	226 (59.16)	12 (3.14)	Nil	140 (36.65)	3 (0.78)	1 (0.26)	348 (91.1)	15 (3.92)	1 (0.26)
Is immunization prohibited by your religion?	26 (6.81)	30 (7.85)	182 (47.64)	26 (6.81)	16 (4.18)	102 (26.70)	98 (25.65)	56 (14.65)	284 (74.34)
Do you think the timing of vaccinations matters?	185 (48.42)	33 (8.63)	20 (5.24)	121 (31.67)	14 (3.66)	9 (2.35)	306 (80.09)	47 (12.29)	29 (7.59)

**Table 3:** Parents’ perceptions regarding immunization services.

Questions	Islamabad			Rawalpindi			Composite		
	Strongly Agree/ Agree *n* (%)	Neutral *n* (%)	Strongly Disagree/ Disagree *n* (%)	Strongly Agree/ Agree *n* (%)	Neutral *n* (%)	Strongly Disagree/ Disagree *n* (%)	Strongly Agree/ Agree *n* (%)	Neutral *n* (%)	Strongly Disagree/ Disagree *n* (%)
Do you think the immunization services offered by the government are satisfactory?	162 (42.40)	31 (8.11)	45 (11.78)	93 (24.34)	26 (6.80)	25 (6.54)	255 (66.74)	57 (14.91)	70 (18.32)
Do you think fear of temporary side effects are a hurdle in adoption of vaccination?	108 (28.27)	49 (12.82)	81 (21.20)	77 (20.15)	30 (7.85)	37 (9.68)	185 (48.42)	79 (20.67)	118 (30.88)
Do you think an awareness campaign about childhood immunization is effective for the community?	214 (56.02)	19 (4.97)	5 (1.30)	121 (31.67)	14 (3.66)	3 (0.78)	335 (87.69)	33 (8.63)	12 (3.13)
Do you think an awareness campaign about childhood immunization is active in Pakistan?	181 (47.38)	25 (6.54)	32 (8.37)	98 (25.65)	20 (5.23)	26 (6.80)	279 (73)	45 (11.77)	58 (15.17)

**Table 4:** Perceptions of parents regarding factors responsible for delay in immunization of their children.

Questions	Islamabad			Rawalpindi			Composite		
	Strongly Agree/ Agree *n* (%)	Neutral *n* (%)	Strongly Disagree/ Disagree *n* (%)	Strongly Agree/Agree *n* (%)	Neutral *n* (%)	Strongly Disagree/ Disagree *n* (%)	Strongly Agree/ Agree *n* (%)	Neutral *n* (%)	Strongly Disagree/ Disagree *n* (%)
Affordability	144 (37.69)	17 (4.45)	77 (20.15)	76 (19.89)	17 (4.45)	51 (13.35)	220 (57.58)	34 (8.9)	128 (33.5)
Unavailability of vaccines	129 (33.77)	35 (9.16)	74 (19.37)	74 (19.37)	13 (3.40)	57 (14.92)	202 (53)	48 (12.56)	131 (34.29)
Non-cooperative attitude of staff	91 (23.82)	47 (12.30)	100 (26.17)	52 (13.61)	34 (8.90)	58 (15.18)	143 (37.43)	81 (21.2)	158 (41.35)
Long waiting times	102 (26.70)	31 (8.11)	105 (27.48)	59 (15.44)	22 (5.75)	63 (16.49)	161 (42%)	53 (13.89)	168 (43.97)
Lack of awareness regarding immunization timing	190 (49.73)	15 (3.92)	33 (8.63)	108 (28.27)	12 (3.14)	24 (6.28)	298 (78)	27 (7.06)	57 (14.91)
Lack of access to immunization centers	133 (34.81)	33 (8.63)	72 (18.84)	91 (23.82)	17 (4.45)	36 (9.42)	224 (58.63)	50 (13.08)	108 (28.26)

**Table 5:** Knowledge of parents regarding immunization and its schedule for their children under two years of age.

Immunization Schedule	Islamabad		Rawalpindi		Composite	
	Correct *n* (%)	Incorrect *n* (%)	Correct *n* (%)	Incorrect *n* (%)	Correct *n* (%)	Incorrect *n* (%)
Vaccine given at birth	26 (6.80)	210 (54.97)	16 (4.18)	128 (33.50)	42 (10.98)	338 (88.46)
Vaccines given at 6 weeks	22 (5.75)	216 (56.54)	18 (4.71)	126 (32.98)	40 (10.46)	342 (89.5)
Vaccines given at 10 weeks	21 (5.49)	217 (56.80)	11 (2.88)	133 (34.81)	31 (8.37)	350 (91.6)
Vaccines given at 14 weeks	17 (4.45)	221 (57.85)	15 (3.92)	129 (33.77)	32 (8.37)	350 (91.6)
Vaccines given at 9 months	27 (7.08)	210 (54.97)	19 (4.97)	125 (32.72)	46 (12)	335 (87.67)
Vaccines given at 12 months	28 (7.33)	210 (54.97)	25 (6.54)	119 (31.15)	53 (13.87)	329 (86.1)
Do you know about the immunization day	81 (21.20)	157 (41.09)	88 (23.03)	72 (18.84)	169 (44.23)	229 (59.93)
Do you know about the age up to which immunization is given to your children	134 (35.07)	104 (27.22)	76 (19.89)	68 (17.80)	210 (54.96)	172 (44.99)
Have you ever experienced any adverse event with your child due to immunization	80 (20.94)	158 (41.36)	61 (15.96)	83 (21.72)	142 (37)	241 (63.07)
Have you ever heard about mobile immunization team	174 (45.55)	64 (16.75)	91 (23.82)	53 (13.87)	265 (69.37)	117 (30.6)

**Table 6:** Parents’ satisfaction with immunization services offered in Pakistan.

Immunization Schedule	Islamabad		Rawalpindi		Composite	
	Correct *n* (%)	Incorrect *n* (%)	Correct *n* (%)	Incorrect *n* (%)	Correct *n* (%)	Incorrect *n* (%)
Are you satisfied with the services provided by a mobile immunization team?	140 (36.64)	98 (25.65)	73 (18.36)	71 (18.58)	214 (56)	168 (44)
Are you satisfied with the immunization provided at hospital centers?	174 (45.55)	64 (16.77)	107 (28.01)	37 (9.68)	282 (73.56)	100 (26.44)
Are immunization services provided to children free of cost?	144 (38.22)	92 (24)	97 (25.39)	47 (12.30)	243 (64)	139 (36)

**Table 7:** Measures to be taken for the improvement of immunization services in Pakistan.

Suggestions	Islamabad Answer ‘Yes’, *n*	Rawalpindi Answer ‘Yes’, *n*	Composite *n* (%)
Promotion of awareness and training of parents regarding the immunization of children	63	22	85 (26.17)
Easy availability and accessibility to immunization centers regulatory enforcement	14	8	22 (5.75)
Regulatory enforcement	6	5	11 (3.40)
Proper vaccine management system/health system	11	8	19 (8.90)
Not satisfied with service	3	2	5 (1.30)
Computerized vaccination system/tele-health/vaccination reminder	5	2	14 (3.91)
Cheaper/free of cost vaccine	27	0	27 (7.06)
Improve the services of the mobile immunization team, the availability, and involvement of the mobile immunization team	12	10	22 (6.54)
Involvement of religious scholars	9	2	11 (2.87)
Cooperative staff	9	6	15 (3.92)
Not responded	43	54	97 (27.22)
Satisfied	6	4	10 (2.61)
Promotion of awareness, improvement of immunization services	10	5	15 (3.92)

**Table 8:** Comparison of parents’ knowledge scores about the immunization of their children under two years of age by demographic characteristics.

Variable			
Gender	*n*	Knowledge Score (14–28) Mean Ranks	*p*-Value
Male	231	195.56	0.201
Female	169	181.77	
City: Islamabad	144	189.35	0.811
Rawalpindi	238	192.80	
Occupation: Government employee	53	146	0.121
Healthcare professional	49	110	
Private employee	74	140	
Businessman	95	155.80	
Engineer	25	141.80	
Academic staff	86	125.95	
Age, y: Less than 20	4	160.62	0.418
Between 20 and 30	166	196.58	
Between 30 and 40	161	181.61	
More than 40	51	202.97	
Qualification: No formal education	7	304.50	**0.002**
Primary education	14	240.49	
Secondary education	59	215.84	
Higher secondary education	20	209.52	
Education up to graduation	145	181.21	
Education up to master’s level	137	177.28	
Monthly income in Rs: Less than 10,000	53	182.77	**0.021**
10,000–20,000	60	175.77	
20,000–30,000	42	131.97	
Rs 30,000–40,000	73	145.42	
40,000–50,000	64	134.90	
More than 50,000	90	138.49	
Number of Children: 1	105	182.99	0.851
2	127	191.29	
3	82	192.85	
4	68	199.71	

Values in boldface indicate *p*-values < 0.05.

## Data Availability

The anonymized datasets used and analyzed during the current study are available from the corresponding author on reasonable request.

## References

[1] Topuzoğlu A., Ay P., Hidiroglu S., Gurbuz Y. (2007). The barriers against childhood immunizations: A qualitative research among socio-economically disadvantaged mothers. Eur. J. Public Health.

[2] Miller N.Z., Goldman G.S. (2011). Infant mortality rates regressed against number of vaccine doses routinely given: Is there a biochemical or synergistic toxicity?. Hum. Exp. Toxicol..

[3] Vashishtha V.M., Kumar P. (2013). 50 years of immunization in India: Progress and future. Indian Pediatr..

[4] WHO Immunization. http://www.who.imt/topics/immunization/en/.

[5] Kalaij A.G.I., Sugiyanto M., Ilham A.F. (2021). Factors Associated With Vaccination Compliance in Southeast Asian Children: A Systematic Review. Asia Pac. J. Public Health.

[6] De Vito E., Parente P., De Waure C., Poscia A., Ricciardi W. (2017). A Review of Evidence on Equitable Delivery, Access and Utilization of Immunization Services for Migrants and Refugees in the WHO European Region.

[7] WHO (2009). State of the World’s Vaccines and Immunization.

[8] Paudyal S. (2013). Knowledge, attitude and practice of immunization behavior among mothers visiting Amda hospital. JHAPA Asian J. Pharm. Sci..

[9] UN Millinium Development Goal. http://www.unmillenniumproject.org/goals/gti.htm.

[10] Baker D.L., Dang M.T., Ly M.Y., Diaz R. (2010). Perception of barriers to immunization among parents of Hmong origin in California. Am. J. Public Health.

[11] Omer S.B., Salmon D.A., Orenstein W.A., deHart M.P., Halsey N. (2009). Vaccine refusal, mandatory immunization, and the risks of vaccine-preventable diseases. N. Engl. J. Med..

[12] Tagbo B.N., Uleanya N.D., Omotowo I.B. (2013). Mothers’ knowledge and perception of Adverse Events following immunization in Enugu, Southeast Nigeria. J. Vaccines Vaccin..

[13] Pakistan Bureau of Statistics Hospitals/Dispensaries and Beds by Province. https://www.pbs.gov.pk/content/hospitalsdispensaries-and-beds-province.

[14] Number of Hospitals and Dispensaries in Punjab. https://pshealthpunjab.gov.pk/.

[15] Raosoft Sample Size Calculator. http://www.raosoft.com/samplesize.html.

[16] Zagminas K., Surkiene G., Urbanovic N., Stukas R. (2007). Parental attitudes towards children’s vaccination. Medicina.

[17] Asim M., Malik N., Yousaf H., Gillani I., Habib N. (2012). An assessment of parental knowledge, belief and attitude toward childhood immunization among minorities in rural areas of district Faisalabad, Pakistan. Mediterr. J. Soc. Sci..

[18] NFHS Child Health. http://rchiips.org/Nfhs/NFHS-3%20Data/VOL-1/Chapter%2009%20-%20Child%20Health%20(562K).pdf.

[19] García L D.A., Velandia-González M., Trumbo S.P., Pedreira M.C., Bravo-Alcántara P., Danovaro-Holliday M.C. (2014). Understanding the main barriers to immunization in Colombia to better tailor communication strategies. BMC Public Health.

[20] Ventola C.L. (2016). Immunization in the United States: Recommendations, barriers, and measures to improve compliance: Part 1: Childhood vaccinations. J. Pharm. Ther. Clin. Risk Manag..

[21] Tefera Y.A., Wagner A.L., Mekonen E.B., Carlson B.F., Boulton M.L. (2018). Predictors and Barriers to Full Vaccination among Children in Ethiopia. Vaccines.

[22] Siddiqi N., Siddiqi A.E., Nisar N., Khan A. (2010). Mothers’ knowledge about EPI and its relation with age-appropriate vaccination of infants in peri-urban Karachi. J. Pak. Med. Assoc..

[23] Angadi M.M., Jose A.P., Udgiri R., Masali K.A., Sorganvi V. (2013). A study of knowledge, attitude and practices on immunization of children in urban slums of bijapur city, karnataka, India. J. Clin. Diagn. Res. JCDR.

[24] Lorenz C., Khalid M. (2012). Influencing factors on vaccination uptake in Pakistan. J. Pak. Med. Assoc..

[25] Raithatha N., Holland R., Gerrard S., Harvey I. (2003). A qualitative investigation of vaccine risk perception amongst parents who immunize their children: A matter of public health concern. J. Public Health Med..

[26] Naeem M., Abbas S.H., Gul S., Adil M., Khan M.Z.-U.-I., Khan M.S. (2012). Causes of immunization failure in DPT vaccination in urban and rural areas of Peshawar. J. Med. Sci..

[27] Mahalingam S., Soori A., Ram P., Achappa B., Chowta M., Madi D. (2014). Knowledge, attitude and perceptions of mothers with children under five years of age about vaccination in Mangalore, India. Asian J. Med. Sci..

[28] Anderson E.L. (2014). Recommended solutions to the barriers to immunization in children and adults. Mo. Med..

[29] Gore P., Madhavan S., Curry D., McClung G., Castiglia M., Rosenbluth S.A., Smego R.A. (1999). Predictors of childhood immunization completion in a rural population. Soc. Sci. Med..

[30] Kiptoo E., Esilaba M., Kobia G., Ngure R. (2015). Factors influencing low immunization coverage among children between 12–23 months in East Pokot, Baringo Country, Kenya. Int. J. Vaccines.

[31] Ismail I.T., El-Tayeb E.M., Omer M.D., Eltahir Y.M., El-Sayed E.T., Deribe K. (2014). Assessment of Routine Immunization Coverage in Nyala Locality, Reasons behind Incomplete Immunization in South Darfur State, Sudan. Asian J. Med. Sci..

[32] Wiysonge C.S., Uthman O.A., Ndumbe P.M., Hussey G.D. (2012). Individual and contextual factors associated with low childhood immunisation coverage in sub-Saharan Africa: A multilevel analysis. PLoS ONE.

